# World health status 1950-2015: Converging or diverging

**DOI:** 10.1371/journal.pone.0213139

**Published:** 2019-03-19

**Authors:** Srinivas Goli, Moradhvaj, Swastika Chakravorty, Anu Rammohan

**Affiliations:** 1 Centre for the Study of Regional Development, School of Social Sciences (SSS), Jawaharlal Nehru University (JNU), New Delhi, India; 2 Department of Economics, The University of Western Australia (M251), Perth, Australia; National Institute of Health, ITALY

## Abstract

**Objective:**

To advance the goal of “Grand Convergence” in global health by 2035, this study tested the convergence hypothesis in the progress of the health status of individuals from 193 countries, using both standard and cutting-edge convergence metrics.

**Methods:**

The study used multiple data sources. The methods section is categorized into two parts. (1) Health inequality measures were used for estimating inter-country inequalities. Dispersion Measure of Mortality (DMM) is used for measuring absolute inequality and Gini Coefficient for relative inequality. (2) We tested the standard convergence hypothesis for the progress in Infant Mortality Rate (IMR) and Life Expectancy at Birth (LEB) during 1950 to 2015 using methods ranging from simple graphical tools (catching-up plots) to standard parametric (absolute β and σ-convergence) and nonparametric econometric models (kernel density estimates) to detect the presence of convergence (or divergence) and convergence clubs.

**Findings:**

The findings lend support to the "rise and fall" of world health inequalities measured using Life Expectancy at Birth (LEB) and Infant Mortality Rate (IMR). The test of absolute β-convergence for the entire period and in the recent period supports the convergence hypothesis for LEB (β = -0.0210 [95% CI -0.0227 - -0.0194], p<0.000) and rejects it for IMR (β = 0.0063 [95% CI 0.0037–0.0089], p<0.000). However, results also suggest a setback in the speed of convergence in health status across the countries in recent times, 5.4% during 1950–55 to 1980–85 compared to 3% during 1985–90 to 2010–15. Although inequality based convergence metrics showed evidence of divergence replacing convergence during 1985–90 to 2000–05, from the late 2000s, divergence was replaced by re-convergence although with a slower speed of convergence. While the non-parametric test of convergence shows an emerging process of regional convergence rather than global convergence.

**Conclusion:**

We found that with a current rate of progress (2.2% per annum) the “Grand convergence” in global health can be achieved only by 2060 instead of 2035. We suggest that a roadmap to achieve “Grand Convergence” in global health should include more radical changes and work for increasing efficiency with equity to achieve a “Grand convergence” in health status across the countries by 2035.

## Introduction

Reduction in mortality reflects improvements in the health and well-being of populations. The brighter side of the global health trends is that there has been an enormous payoff from investing in health in the last two decades and a half [1]. Human life is superior now compared to any time in the past. The ‘great escape’ from the mortality trap [2] that prevailed until the early 19^th^ century has led to the world’s population now living longer and healthier lives than at any other time in the past [3]. The life expectancy of the population has progressed, not only at the time of birth but also across all age groups and across different countries [4,5]. The declines in mortality rates, especially during childhood, have been particularly remarkable [4,6,7]. Globally, life expectancy at birth (LEB) has increased from 46.5 years in 1950–55 to 65.0 years in 1995 to 2000, and to 75.0 years in 2010–15, accounting to more than 50% improvement from 1950–55 to 2010–15. Over the same period, the number of years that a newborn is expected to live, on average, increased globally by 24 years, or by about 3.6 years per decade [8]. However, the dark side is the persistence of health inequalities across the regions and within individual countries.

While the between-country health inequality in the world is well known [5,6,7,9], how far the differences are converging, or diverging and its correlates have been a matter of great interest for health policy and monitoring. The debate on between-country mortality rates and health inequalities continue to be largely debated based on the analyses of the recent trends or the current levels [5,6,10,11]. However, analyses based on recent mortality and health data have serious limitations in understanding the true trajectories of between-country inequalities [12]. With changing socioeconomic, demographic and epidemiological scenarios across countries[3,13], the major concern for global health policy has been the heterogeneity and variation in the tempo of health transition across regions, and countries within the regions in the wake of diverging economies, health systems, and healthcare spending.

This study looks at how health is faring worldwide and focuses on providing appropriate metrics for measuring and monitoring health policies and indicators. We aim to use a set of econometrics tools to study the mortality and health transition, convergence and its correlates. The study advances the empirical examination of summary measures of convergence assessment by investigating convergence not only in the averages but also in the absolute and relative inequalities in key health indicators and its correlates.

### Previous evidence

Despite significant progress in average mortality indicators throughout the world, researchers are still divided in charting whether world-wide improvement were accompanied by a deterministic process of convergence in population health. For instance, McMichael and colleagues [14]. analyzed mortality data from 1950 to 2000 and drew attention to three groups of countries based on life expectancy trends: those that have experienced rapid improvements in their mortality experience and life expectancy, those that have experienced relative stagnation, and those that have experienced an erosion of life expectancy and reversals in mortality. Others such as Moser and colleagues [15] and Neumayer [16] demonstrated that despite showing an overall upward trajectory, the emerging picture of erratic mortality trends and regional setbacks indicated that future health gains are not guaranteed by any general deterministic process of convergence. Among others who empirically tested the convergence hypothesis on long-term mortality and health data have reached diverse conclusions. A majority of these studies find that convergence was replaced with divergence since the late 1980s [17,18,19,20]. Wilson [18] supported convergence hypothesis, while Bloom and Canning [2], Dorius [12] and Coal and Niemeyer [17], Goli and Arokiasamy [21] suggested the rising of convergence clubs rather than absolute convergence; and also, greater divergence in life expectancies from the 1990s.

Previous literature has documented empirical evidence on convergence [or divergence] hypotheses on trends in world health status until 2005 [12,14,15,18]. While the most recent research on trends in health status adopts an optimistic perspective, which aims for a “Grand convergence” in health across the world within a lifetime. Although the recent literature suggests the need for analyzing opportunities and challenges in achieving convergence, it has not specifically tested the convergence hypothesis empirically [20,22]. In particular, based on existing evidence [12,14,15,18] we are unable to assess the speed at which countries across the world are converging in terms of their health status.

## Data sources

The study used multiple data sources. We have compiled data on Infant mortality rates (IMR) and Life expectancy at birth (LEB) from United Nations’ Population Prospects for all 193 countries with a population of at least one million in 2015. We have obtained data on other socio-economic, health outcome and health care, household environment and climatic covariates for all the select countries. We consider literacy and Gross Domestic Product (GDP) per capita as indicators of social and economic development, while IMR, LEB, Maternal Mortality Ratio (MMR) and per capita health care expenditure measures of health outcomes and health care. Access to improved water and sanitation were considered as indicators of household environment, while Carbon dioxide (CO2) emissions were included as an indicator of a macroclimatic factor. Details of the data sources are described in Table 1.

**Table 1:** List of Indicators used and details of the data sources.

*Indicator*	*Data Source*
Infant Mortality Rate (Per 1000 live birth)	United Nations Population Division, World Population Prospects from 1950–55 to 2010–15.) (http://data.un.org/Data.aspx?q=Infant+mortality+rate+1950&d=PopDiv&f=variableID%3a77%3btimeID%3a2
Life Expectancy at Birth (Year)	United Nations Population Division, World Population Prospects form 1950–55 to 2010–15) (http://data.un.org/Data.aspx?q=Life+expectancy&d=PopDiv&f=variableID%3a68
Literacy rate (%)	UIS data centre, UNESCO (http://data.uis.unesco.org/Index.aspx?DataSetCode=EDULIT_DS&popupcustomise=true&lang=en#)
Total Population (Number)	United Nations Population Division, World Population Prospects for 2015 (http://data.un.org/Data.aspx?q=total+population+2015&d=PopDiv&f=variableID%3a12%3btimeID%3a79)
MMR (Per 100,000 live birth)	World Health Organization, 2015 (http://www.who.int/gho/maternal_health/countries/en/)
Health expenditure per capita (US$)	World Health Organization Global Health Expenditure database for 2015 compiled by the World Bank (http://apps.who.int/nha/database/Select/Indicators/en)
GDP Per capita (US$)	World Health Organization Global Health Expenditure database for 2015 compiled by the World Bank (http://apps.who.int/nha/database/Select/Indicators/en)
Improved water source (%)	WHO/UNICEF Joint Monitoring Programme (JMP) for Water Supply and Sanitation (https://data.worldbank.org/indicator/SH.H2O.SAFE.ZS)
Improved sanitation facilities (%)	WHO/UNICEF Joint Monitoring Programme (JMP) for Water Supply and Sanitation (https://data.worldbank.org/indicator/SH.STA.ACSN)
CO2 emission (Metric tons per capita)	Carbon Dioxide Information Analysis Center, Environmental Sciences Division, Oak Ridge National Laboratory, Tennessee, United States 2015 compiled by the World Bank(https://data.worldbank.org/indicator/EN.ATM.CO2E.PC), Emissions Database for Global Atmospheric Research (http://edgar.jrc.ec.europa.eu/overview.php?v=CO2ts_pc1990-2015)
Antenatal Care (ANC) visits (% at least one)	UNICEF (https://data.unicef.org/topic/maternal-health/antenatal-care/)
Children full immunization (%)	WHO and UNICEF (http://www.who.int/gho/immunization/en/) and (https://data.unicef.org/topic/child-health/immunization/)

## Methodology

### I. Measuring health inequality

The paper reflects on the debate of measuring health inequalities as distinct from measuring the average levels of health. The analyses in this paper are presented in two parts. The first part mainly deals with estimating inter-country inequalities in health, and the second part deals with whether the improvements in health indicators or health gaps are converging or diverging. In the first part, the global health inequalities at a point in time were quantified by using the Dispersion Measure of Mortality (DMM) and the Gini Coefficient. The trends in the DMM and Gini Coefficient indicate absolute and relative inequalities respectively. The mathematical proofs of the models are given below.

#### 1.1 Dispersion Measure of Mortality

The DMM quantifies the degree of dispersion that exists at a given point of time in the mortality experiences of a particular country. It is calculated as the average of the absolute difference in mortality, weighted by population size, between every pair of countries. Changes in the DMM over time indicate whether mortality is becoming more or less similar across the world. Thus, a decrease indicates convergence and an increase indicates divergence.

The DMM for life expectancy at birth is measured using years of life, and the DMM for infant mortality is measured in infant deaths per thousand live births. It is obtained by the formula given below [15, 23].

$$DMM=\frac{1}{{2(W}_{Z}{)}^{2}}\sum_{i}\sum_{J}\left({|M}_{i}-M_{j}|*W_{I}*W_{J}\right)$$

Where, *I*, *j* are countries, and 1 ≤*i*, *j*≤ 193

Z is equal to 1

M is the mortality rate

W is the weighting and ∑_i_W_i_ = ∑_j_W_j_ = W_z_

When applied to life expectancy at birth, M = life expectancy at birth of the country, W_z_ = 1 and W_I_ represents the relative population size of the country. The DMM claims to have an advantage over other commonly used measures as it not only uses information about mortality and socio-economic distribution but also provides weight for the size of the unit.

#### 1.2. Gini coefficient

To assess the overall relative inequality, this study used the Gini concentration index. The Gini coefficient is an extremely informative measure since it examines all parts of the distribution at once. In case of the quantitative variable, it also facilitates direct comparisons of two or more populations, regardless of their sizes. It can, therefore, be used efficiently for the comparison of inequality between groups, countries or regions

The estimation of Gini for Life Expectancy at Birth (LEB) is equal to DMM divided by the average life expectancy of the countries [10].

$$G=\frac{DMM}{\bar{e_{0}^{0}}},where\;\bar{e_{0}^{0}}=\left[\sum_{i}Pi\;e_{0}^{i}\right]$$

Where G = Gini index value, DMM = Dispersion Measure of Mortality, $\bar{e_{0}^{0}}$ is average life expectancy at birth adjusted by the population proportion of the country i…i_n_.

### II. Convergence metrics

#### 2.1 Absolute β-convergence model

The absolute β-convergence measure is used where the gap between laggard and developed nations shrinks especially due to higher progress in laggard nations. β-Convergence model in a cross-section of nations assumes a negative relationship between the growth rate of an indicator and its initial levels. It is estimated by a regression model proposed by Barro [24].

This model is represented in the form of the following equation:

$$In\left[\frac{Y_{i,t+k}}{Y_{i,t}}\right]=\alpha +\beta .\ln\left(y_{i.t}\right)+\varepsilon_{it}$$

Where $In\left[\frac{Y_{i,t+k}}{Y_{i,t}}\right]$ is the mean annualized growth rate of the variable y in the state *i* in the period (t, t+k), *y*_*i*.*t*_ is the value in the initial time t and *ε*_*it*_ are the corresponding residuals.

Further, the speed of convergence is computed as: *s* = − [ln (1+*T*β) /*T*]. Where the term s = speed of convergence and *T*β is the *β-convergence* in *T* period.

Based on the speed of convergence, the time to reach absolute convergence is estimated as:

T = [Range in LEB in 2015/ (Range in LEB in 2015*(speed of convegence in 1950 to 2015/100)].

*β-Convergence* is a necessary but not sufficient condition for σ-convergence. Thus, it is imperative to test σ- convergence alongside *β-Convergence* [25].

#### 2.2 σ -convergence measure

A reduction in the cross-sectional dispersion of a variable over time indicates σ- convergence. Σ- convergence measure postulates that convergence occurs when the dispersion of health indicators decreases [25]. Extending this logic to the case of health inequalities around the world, we expect the standard deviation between health indicators (LEB and IMR) across the nations to shrink eventually. The σ -convergence model was estimated as:

$$\sigma_{t}>\sigma_{t+T}$$

Where *σ*_*t*_ is the standard deviation (or assimilated measure) of the indicator at the time *t*. If the parameter *σ*_*t*+*K*_ reduces, it implies evidence of convergence.

Although the measure of convergence through standard deviation by taking the health outcome measures in their original form can be problematic in the case of changing means, standard deviations move according to movement in the mean of the health measures with the constant distributional pattern. However, alterations in the method such as taking variables in its original form or taking logarithmic of the health measures will hardly make any significant difference for the analysis of convergence trends through standard deviation [16].

#### 2.3 Kernel density estimator

Similar to the income transition story, health transition is also characterized by the existence of dual regimes of high and low mortality [26]. Over time, as convergence prevails, the second peak is predicted to disintegrate since all the countries adhere to the longer life and fewer deaths regime. Quah [21] points out that the idea underlying the regressions of growth on the initial level of the indicator is that the initial condition determines the transitory dynamics, while the conditioning variables explain the trend. However, the underlying assumption that every country has a stable growth path well-approximated by a time trend may not always stand true. Unlike the parametric measures, non-parametric approaches do not rely on assumptions about the nature of data except perhaps its smoothness [27, 28], thus enabling them to present a closer picture regarding convergence.

The kernel estimator is one of the most widely used non-parametric techniques to study convergence [23]. It may be defined as

Let *f* = *f (x)* denote the continuous density function of a random variable *X* at a point *x*, and let *x1*, …,*xn be* the observations from *f*.

Rosenblatt [24] defined a kernel function *K* as:

$$\int_{-\infty }^{\infty }K(y)dy=1$$

Where, *K*(*y*)≥0.

The general kernel estimator *f*ˆ (*x*) is defined by:

$$\hat{f(x)}=\frac{1}{nh}\sum_{i=1}^{n}K\left(\frac{x_{i}-x}{h}\right)=\frac{1}{nh}\sum_{i=1}^{n}K(Yi)$$

Where, *Y*_*i*_ = *h*^−1^(*x*_*i*_−*x*), *n* is the number of observations in the sample, *h* is the window width (bandwidth) which is a function of the sample size and goes to zero as n→∞.

### Convergence metrics

There is no harmonisation among scholars on the process and measures of convergence. Therefore, this study used all available convergence metrics to draw the most agreeable conclusion on the test of convergence hypothesis on progress in trends of global health status. The convergence process was examined using methods ranging from simple graphical tools (catching-up plots) to standard parametric (absolute β and σ-convergence), and nonparametric econometric models (kernel density estimates) to detect the presence of convergence (or divergence) and convergence clubs. Previous studies [2,12,14,15,19] which adopted these statistical tools considered them as being novel approaches to the measurement of global health convergence in their respective studies.

## Results

### LEB and IMR: Trend analysis

We examined the long-term trends in LEB and IMR based on available data for 193 countries for the period, 1950–55 to 2010–15. The results show a 40 percent gain in average LEB and 79 percent reduction in IMR over the study period. However, the range of LEB followed a sinuous pattern, *i*.*e*., fall-rise-fall over the study period, and the range of IMR shows a substantial decline, notwithstanding a considerable increase during 1975–80. The fluctuation in minimum values and the range of LEB indicates more unstable progress in health indicators across the less developed countries, while a stable increase in the maximum LEB reveals that a more consistent trend in the improvement of health status in more developed countries (Table 2).

**Table 2:** Summary statistics of LEB and IMR across world countries, 1950–55, 2010–15.

Year	Observation			Mean			Min		Max		Range	
	LEB	IMR	LEB	CI [95%] (Lower-Upper limit)	IMR	CI [95%] (Lower-Upper limit)	LEB	IMR	LEB	IMR	LEB	IMR
1950–1955	193	193	50.05	48.32–51.78	130.02	120.87–139.16	26.96	20	72.66	281	45.7	261
1955–1960	193	193	52.70	50.96–54.43	114.90	106.17–123.63	27.98	17	73.49	256	45.51	239
1960–1965	193	193	55.03	53.32–56.75	101.60	93.34–109.85	28.61	16	73.55	251	44.94	235
1965–1970	193	193	57.10	55.46–58.75	89.47	81.75–97.19	30.8	13	74.05	216	43.25	203
1970–1975	193	193	59.03	57.45–60.61	78.89	71.67–86.10	34.2	11	74.76	199	40.56	188
1975–1980	193	193	60.80	59.22–62.37	70.34	63.08–77.60	14.49	8	76.23	320	61.74	312
1980–1985	193	193	62.68	61.24–64.12	60.80	54.49–67.12	39.45	6	76.95	184	37.5	178
1985–1990	193	193	64.24	62.84–65.64	53.60	47.69–59.52	39.13	5	78.51	158	39.38	153
1990–1995	193	193	65.09	63.65–66.54	49.13	43.04–55.23	23.05	4	79.45	289	56.4	285
1995–2000	193	193	66.06	64.64–67.48	42.48	37.35–47.61	36.69	4	80.48	149	43.79	145
2000–2005	193	193	67.30	65.86–68.74	36.66	32.08–41.25	40.73	2	81.83	135	41.1	133
2005–2010	193	193	69.09	67.76–70.42	31.28	27.30–35.27	44.96	2	82.62	117	37.66	115
2010–2015	193	193	70.74	69.53–71.96	26.75	23.27–30.23	49.19	2	83.73	96	34.54	94

Note: LEB—Life Expectancy at Birth, IMR—Infant Mortality Rate, SD—Standard Deviation, Min.—Minimum, Max.—Maximum, Confidence Interval (95%) in parentheses

**Fig 1:**
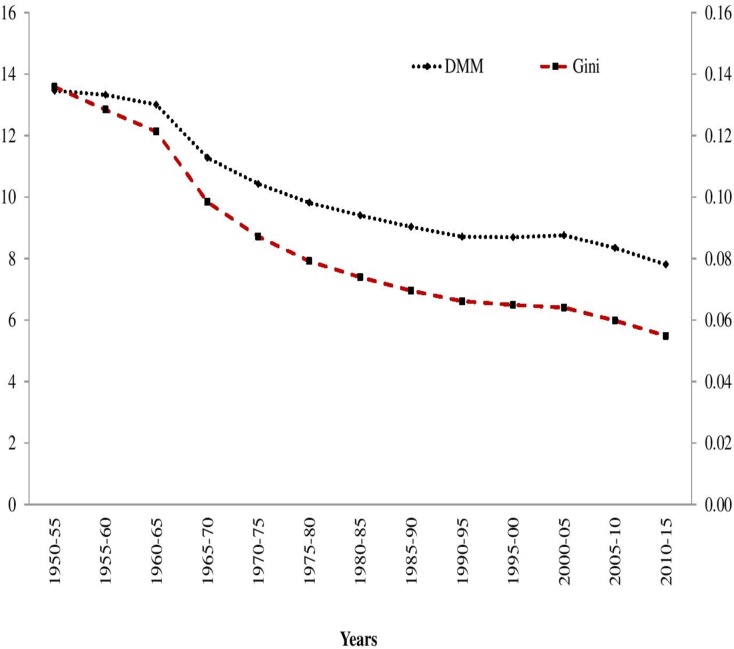
Trends in Dispersion Measure of Mortality (DMM),and Gini Index of LEB across 193 Countries, 1950–55 to 2010–15.

The absolute and relative inequality in both the indicators have been measured using DMM and Gini coefficients respectively. Absolute inequality in LEB shows a continuous declining trend despite post-1990s setbacks that resulted in an acceleration in the rate of reduction. The relative inequality in LEB also shows a similar trend, albeit with a higher rate of reduction. The results of LEB trend for all the three measures affirm a falling health gap across countries during the period, 1950–55 to 2010–2015 (Fig 1).

**Fig 2:**
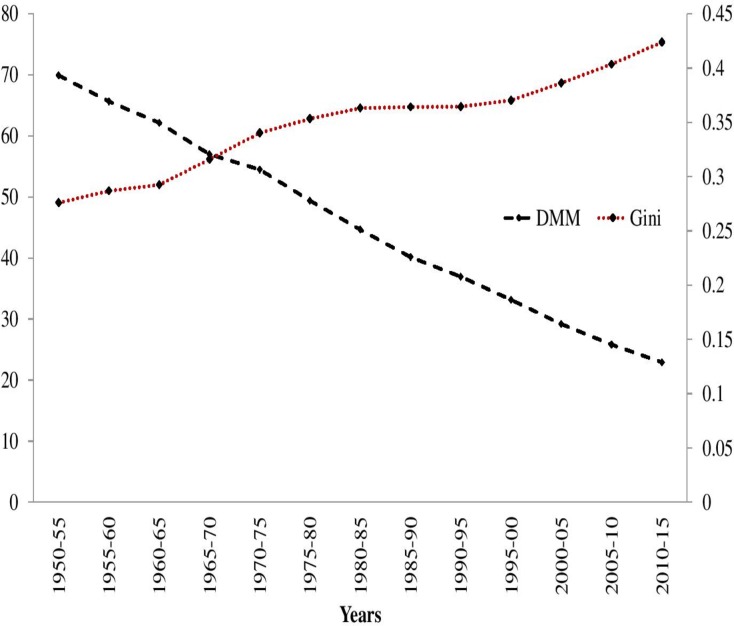
Trends in Dispersion Measure of Mortality (DMM) and Gini Index of IMR across 193 Countries, 1950–55 to 2010–15.

In the case of IMR, the absolute inequality (DMM) showed a continuous decline with the former declining comparatively faster than the latter over the study period. On the other hand, relative inequality (Gini coefficient) in IMR shows an increasing trend, characterised by faster increment during 1960–70 followed by a substantial decline during 1980–95. However, the relative inequality in the last decade shows an increasing trend. The contradiction in the direction of DMM, Gini Coefficient of IMR proves that worldwide, absolute inequality in IMR was on the decline, while relative inequality in IMR continues to rise (Fig 2).

### Testing the convergence hypothesis

#### Convergence: Catching-up process

**Fig 3:**
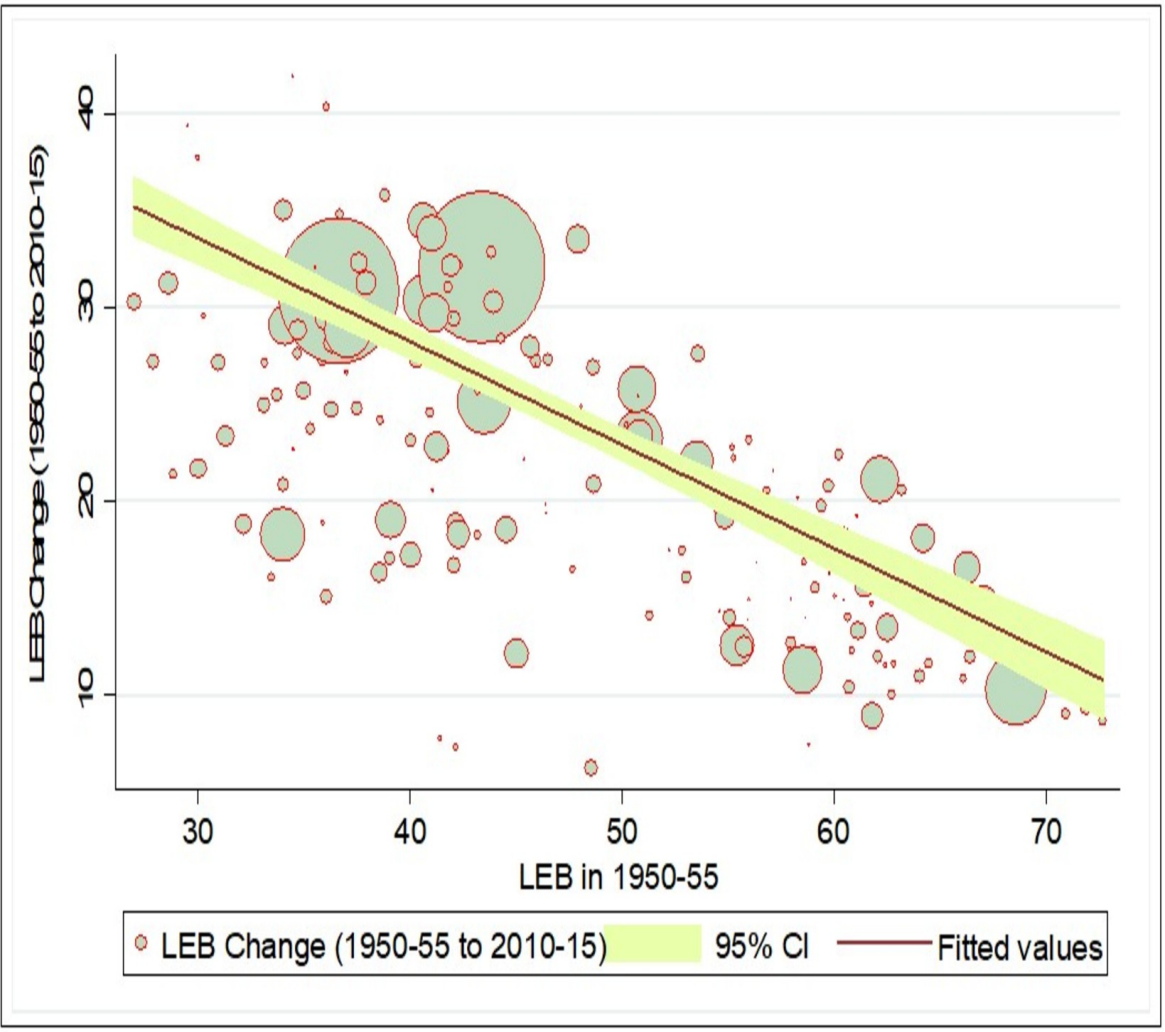
Catching-up process of life expectancy at birth (LEB) across countries, 1950–55, 2010–15.

**Fig 4:**
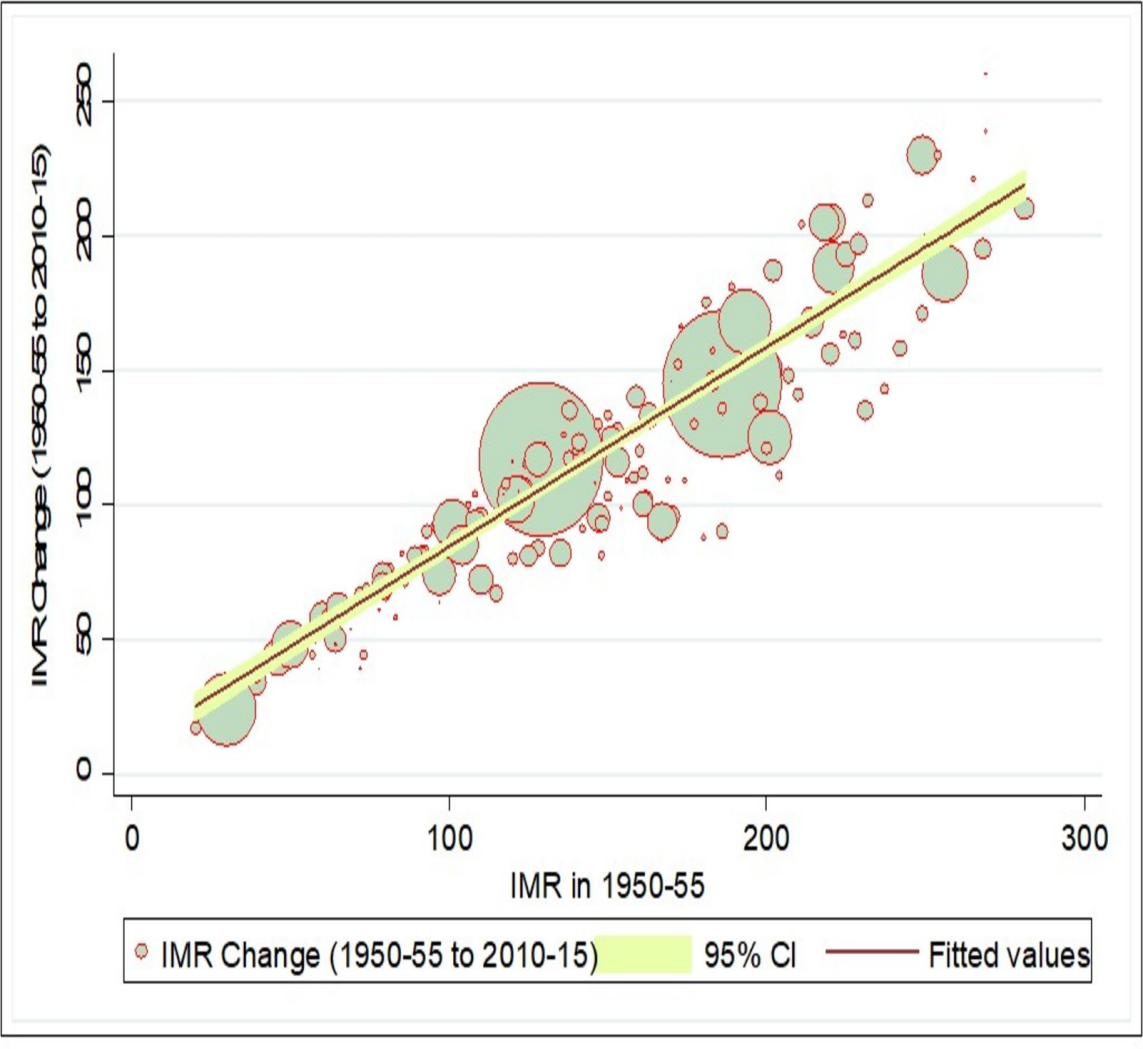
Catching up process of infant mortality (IMR) across countries, 1950–55, 2010–15.

The Neo-classical or Solow’s growth framework^24^ suggested that catching-up mechanism is necessary for convergence, which can be identified by plotting a scatter diagram for changes in an indicator at two points in time (current period) against values in the initial period. Fundamentally, in case of convergence, countries with lower levels of initial LEB should experience a greater gain vis-a-vis lower gain in countries with higher levels of initial LEB. Fig 3 shows the relative change in LEB for two points of time against the mean values in initial period for 193 countries. In case of LEB, our results showed a negative association between change and initial level of LEB. This indicates that the catching-up process is evident for laggard countries in case of LEB, showing that the laggard countries are experiencing higher growth in LEB compared to advanced countries. However, the catching-up process in the LEB is not very strong as still few countries with lower LEB levels experience smaller change, while some countries with higher LEB levels experience higher change during 1950 to 2015. Similarly, Fig 4 indicates that the laggard countries with high levels of IMR in the initial period are experiencing a greater change in IMR compared to the advanced countries with the lower initial level of IMR. Again, although there is evidence of a catch-up process in reduction in IMR in laggard countries, it not very strong.

#### Absolute β−Convergence

Although the catching-up mechanism provides some indication of the emerging convergence process, the volume of convergence can be identified only through classical summary measures of convergence. Table 3 shows the results of the absolute *β*-convergence model for LEB for 193 countries. The results indicate that during the period 1950–55 to 2010–15, progress in LEB shows a statistically significant convergence across countries (*β* = -0.0210, p<0.01). Similarly, the result from piece-wise *β*−convergence model showed the consistent trend of convergence in LEB for the period from 1950–55 to 1980–85 (*β* = -0.0267, p<0.01) and 1985–90 to 2010–15 (*β* = -0.0212, p<0.01). The results support the findings from the catching-up plots, which show that countries with lower initial values of LEB showed higher progress vis-a-vis advanced countries. The results for the speed of convergence estimates revealed that the countries are converging at the rate of 2.2 per year during 1950–2015; speed of convergence is slowing down in the recent decade. The speed of convergence indicates 0.35 years of decline in the range of LEB across countries. With this rate, from 2015 onwards it would take more than 45 years to achieve absolute global convergence in LEB across countries.

**Table 3:** Absolute *β* convergence for life expectancy at birth across countries, 1950–55, 2010–15.

Period	β coefficient	CI [95%] (Lower-Upper limit)	P value	Adjusted R^2^	Speed of convergence (% per annum)
1950–55 to 1980–85	-0.0267	(-0.0294–0.0239)	0.000	0.66	5.4
1985–90 to 2010–15	-0.0212	(-0.0249–0.0175)	0.000	0.40	3.0
1950–55 to 2010–15	-0.0210	(-0.0227–0.0194)	0.000	0.77	2.2

Table 4 showed *β*-convergence for progress in reduction of IMR across the 193 countries showed a significant diverging trend. The result of absolute *β*−convergence showed the same picture as catching-up plots, that in spite of the considerable reduction in the level of IMR during the period 1950–55 to 2010–15, absolute *β*−convergence for IMR shows statistically significant divergence (*β* = 0.0063, p<0.01). Similar findings are observed through the piece-wise *β*-convergence model, which indicated that the rate of reduction in IMR among countries with higher initial values tend to show slower improvement, whereas the rate of reduction is higher among the advanced countries with low level of initial value of IMR. The speed of divergence revealed that the progress in reduction of IMR across the countries is diverging at the rate of -0.5 per year during 1950–55 to 2010–15, which is increasing in the more recent period.

**Table 4:** Absolute *β* convergence for infant mortality rate across countries, 1950–55, 2010–15.

Period	β coefficient	P value	CI [95%] (Lower-Upper limit)	Adjusted R^2^	Speed of convergence (% per annum)
1950–55 to 1980–85	0.0094	0.000	(0.0063–0.0124)	0.16	-0.8
1985–90 to 2010–15	0.0099	0.000	(0.0049–0.0149)	0.07	-0.9
1950–55 to 2010–15	0.0063	0.000	(0.0037–0.0089)	0.10	-0.5

#### σ–Convergence

**Fig 5:**
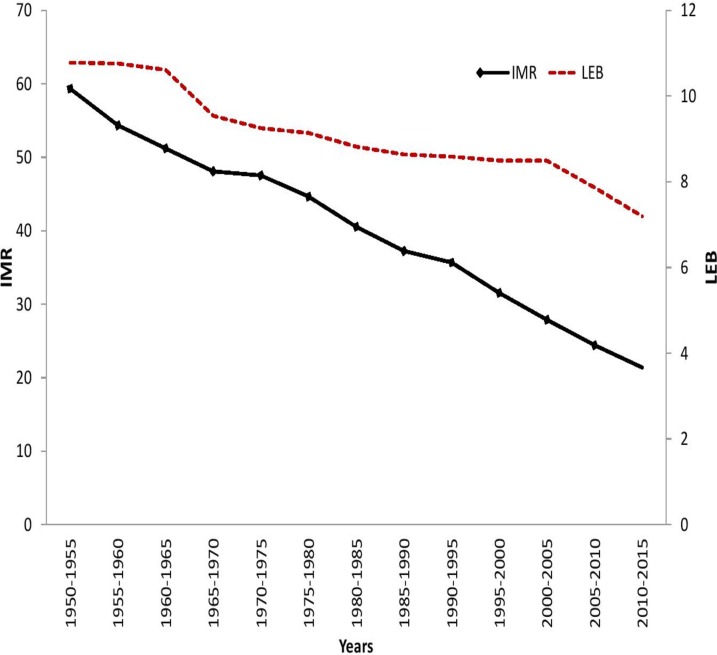
Sigma convergence for life expectancy at birth and infant mortality rate across the countries, 1950–55, 2010–15. Note: estimated based on population-weighted standard deviation.

σ–convergence has been measured using population-weighted standard deviation in LEB and IMR over the sample period and is presented in Fig 5. The trends in standard deviation for both variables show overall convergence at a varied pace. The trends in standard deviation for LEB and IMR showed a constant decline over the period, 1950–2015, suggesting that there is clear evidence for convergence. The rate of convergence in standard deviation had shown a faster decline in recent period notwithstanding the setbacks of post-1990s which stalled or reversed the tide of grand convergence.

#### Convergence clubs: Kernel density estimate

**Fig 6:**
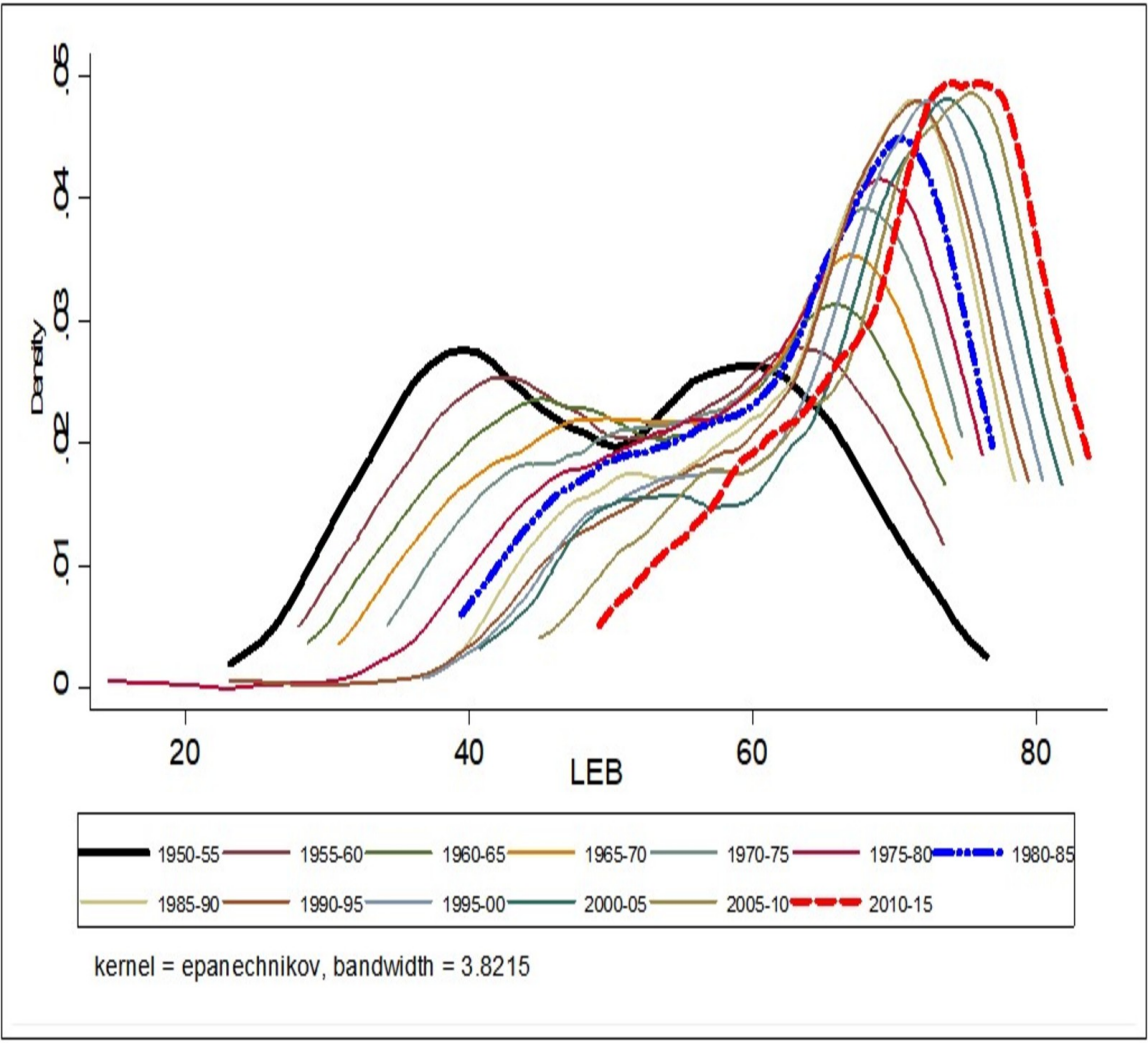
Non-Parametric test (Kernel density plots) of convergence in life expectancy at birth (LEB) across world countries, 1950–55, 2010–15.

**Fig 7:**
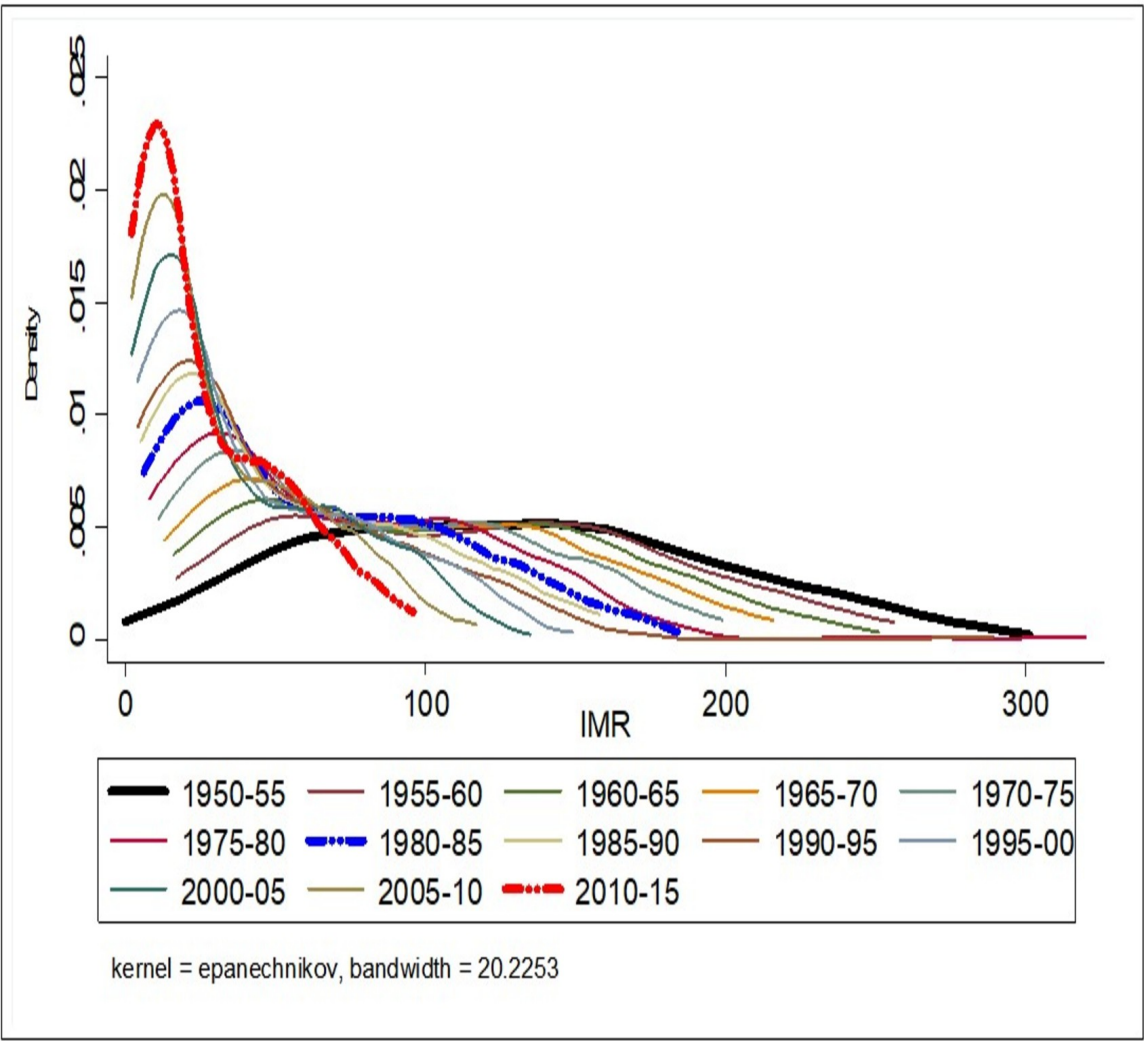
Non-Parametric test (Kernel density plots) of convergence in infant mortality rate (IMR) across world countries, 1950–55, 2010–15.

The findings from testing the hypothesis of convergence clubs through non-parametric tests (Gaussian kernel density plots) indicated the presence of convergence clubs in the case of LEB over the study period. These results are presented in Fig 6. With regards to LEB, both for the period 1950–55 and the more recent 2010–15, we observe evidence of the presence of twin peaks in the Kernel distribution of LEB across the countries. In the period 2010–15, the peak at higher LEB values holds the maximum number of countries which indicates convergence in LEB across countries. This can be substantiated by looking at the spread of peaks, showing the larger spread in peaks for the period 1950–55 compared to peaks for the year 2010–15. While in the case of IMR, the presence of convergence clubs is not evident in 1950–55, but the emergence of convergence clubs can be observed from 2010–15. The result shows that there is evidence of the larger peak at lower IMR values and a smaller secondary peak at higher IMR values, which mean that the largest groups of countries are located under larger peaks with lower IMR, indicates convergence in the year, 2010–15 (Fig 7).

### Correlates of LEB and IMR

There has been ample research into the nature of differences in health transition across countries and regions over time and its causes mostly with micro-level data. Even small differences in the improvement of health, if cumulated over a long period, may have a substantial macro-level impact. Despite considerable research on the subject and global health policy efforts, cross-country and cross-regional health disparities have increased over time. Understanding the determinants of between-country health inequalities is essential to formulate appropriate policies, draw pathways for convergence and bring about required institutional changes to reduce the health inequalities across the world countries. The ideal method to identify the pathways in macro-level analyses is to perform panel data regression models, but due to missing information for a considerable number of predictors for the initial period, we decided to model regressions at a point in time.

We used the OLS regression technique to identify the socio-economic, health outcome and health care, household environment and climatic correlates of LEB and IMR across our sample of countries. We consider literacy and GDP per capita as indicators of social and economic development respectively, while IMR, MMR and per capita health care expenditure as measures of health status outcome and health care. Access to improved water and sanitation were considered as indicators of household environment, while CO2 emissions were included as an indicator of macroclimatic factors. Three separate OLS models were estimated to see the gross influence of socio-economic, health and environmental dimensions separately, while Model-4 was used to see the net influence of all three dimensions together on LEB and IMR. The logarithmic values of dependent and independent values were used in the analyses to reduce skewness and ensure normality of the error terms. Table 5 shows the correlates of LEB across countries for the period 2010–2015. The model 1 shows the effect of literacy and GDP per capita on LEB. The results indicate that literacy (β = 0.1496, p<0.01) and GDP per capita (β = 0.0437, p<0.01) are positively associated with LEB, meaning the country with high levels of literacy rate and GDP per capita have high levels of LEB. Model 2 shows the effect of IMR, MMR and per capita health care expenditure on LEB. The results show that IMR (β = -0.0522, p<0.01) and MMR (β = -0.0335, p<0.01) were negatively associated with LEB, while per capita health care expenditure was positively associated with LEB. In other words, countries with higher levels of IMR and MMR have lower levels of LEB, while countries with higher spending n health care have longer LEB. Model 3 shows a significant positive association between access to improved water (β = 0.1821, p<0.01) and sanitation (β = 0.1195, p<0.01) and LEB, implying that higher access to improved water and sanitation facility is significantly contributing to higher levels of LEB. When we considered all the parameters together in Model 4, the results show that literacy rate, GDP per capita are positively associated with LEB, while IMR (β = -0.0671) was significantly and negatively associated with LEB. Further, countries with higher access to improved water (β = 0.1058, p<0.01) and sanitation facility (β = 0.0609, p<0.01) continue to hold a high positive association with LEB even after controlling for other factors.

**Table 5:** Results of linear regression: Correlates of Life Expectancy at Birth across the countries, 2010–2015, (n = 174).

	Variables	β-Coefficient			
		Model-1	Model-2	Model-3	Model-4
Socio-economic	Log of Literacy rate	0.1496***			0.0081
		(0.0848–0.2143)			(-0.0491–0.0652)
	Log of GDP Per capita	0.0437***			-0.0115
		(0.0285–0.0589)			(-0.0344–0.0114)
Health & Health Care	Log of IMR		-0.0522***		-0.0671***
			(-0.0756–0.0288)		(-0.0986–0.0356)
	Log of MMR		-0.0335***		-0.0096
			(-0.0482–0.0188)		(-0.0311–0.0120)
	Log of Health expenditure per capita (current US$)		0.0023		-0.0002
			(-0.0092–0.0139)		(-0.0241–0.0236)
Household environment and basic amenities	Log of Improved water source			0.1821***	0.1058**
				(0.0930–0.2712)	(0.0200–0.1917)
	Log of Improved sanitation facilities			0.1195***	0.0609***
				(0.0899–0.1492)	(0.0246–0.0971)
Climate	Log of CO2 emission			0.0016	-0.0026
				(-0.0003–0.0035)	(-0.0182–0.0130)
	Constant	3.2103***	4.5154***	2.9301***	3.8114***
		(2.9878–3.4328)	(4.4034–4.6273)	(2.6080–3.2522)	(3.3577–4.2650)
	Prob> F	0	0	0	0
	R-squared	0.6107	0.8554	0.663	0.8705
	Adj R-squared	0.6047	0.8514	0.657	0.859
	Root MSE	0.6246	0.3717	0.6441	0.338

Standard error in parenthesis

**significant at p<0.05

***significant at p<0.01, CO2 emission is proxy of climate change, Confidence Interval (95%) in parentheses.

Table 6 presents the OLS regression results on the association between IMR and adult literacy rate, GDP per capita, maternal health care, and level of child full immunization, access to safe drinking water, sanitation facility and CO2 emissions across our sample of countries. Model 1 shows that countries with high levels of literacy (β = -0.6873) and GDP per capita (β = -0.4748) experience low levels of IMR. Model 2 shows that utilization of ANC care was significantly conducive in reducing IMR across the world. MMR (β = 0.4229, p<0.01) was positively associated with IMR; indicating that countries with poor maternal healthcare facilities have high levels of IMR. Further, countries spending more on health (β = -0.1832, p<0.01) have low IMR. Model 3 shows that access to improved source of drinking water (β = 1.3288, p<0.01) and sanitation (β = 0.3385, p<0.01) facility was negatively associated with IMR. When Model 4 considers all the factors, the results show that literacy rate, GDP per capita and utilization of ANC facilities were negatively associated with IMR. Similarly, countries spending more on health care expenditure continued to show low IMR (β = -0.1499, p<0.045), while countries with poor maternal healthcare services (0.5155) have a high IMR even after controlling for other factors.

**Table 6:** Results of linear regression: Correlates of IMR across the countries, 2010–2015 (n = 174).

	Variables	β-Coefficient			
		Model-1	Model-2	Model-3	Model-4
Socio-economic	Log of Literacy rate	-0.6873***			0.0692
		(-1.1551–0.2196)			(-0.4473–0.3090)
	Log of GDP Per capita	-0.4748***			0.037
		(-0.5847–0.3650)			(-0.1746–0.1006)
Health & Health Care	Log of ANC visit (at least one)		0.1636		0.1976
			(-0.6457–0.3184)		(-0.7241–0.3289)
	Log of Immunization		0.0398		0.0153
			(-0.3481–0.2685)		(-0.3901–0.4206)
	Log of MMR		0.4229***		0.5155***
			(0.3515–0.4944)		(0.4274–0.6037)
Household environment and basic amenities	Log of Health expenditure per capita		0.1832***		0.1499**
			(-0.2580–0.1083)		(-0.2897–0.0101)
	Log of Improved water source			1.3288***	0.4495
				(-2.1117–0.5458)	(-1.0265–0.1276)
	Improved sanitation facilities			0.3385**	0.1638
				(-0.6419–0.0352)	(-0.0659–0.3934)
Climate	Log of CO2 emission			0.3306***	0.0960**
				(-0.4278–0.2334)	(0.0006–0.1914)
	Constant	9.9635***	3.1413***	10.3329***	4.3736
		(8.3566–11.5705)	(0.8451–5.4375)	(7.3049–13.3609)	(1.3587–7.3884)
	Prob> F	0	0	0	0
	R-squared	0.6107	0.8554	0.663	0.8705
	Adj R-squared	0.6047	0.8514	0.657	0.859
	Root MSE	0.62463	0.3717	0.6441	0.338

Standard error in parenthesis

**significant at p<0.05

***significant at p<0.01, MMR is proxy of maternal healthcare; CO2 emission is proxy of climate change, Confidence Interval (95%) in parentheses.

## Discussion

In this study, we used both standard and inequality based convergence metrics to test the hypothesis of convergence both in averages and relative distribution of LEB and IMR in 193 countries worldwide. Our results confirm the findings of previous studies [1,12,14,15,16,18,20] that found evidence of considerable progress in global averages of LEB and IMR. Our findings revealed a trend of ‘rise-fall-rise’ in inequalities in world health status during the period 1950 to 2015, despite the fact that the speed of fall being slow in the recent times. The progress in relative and absolute inequality for LEB is found to be in the direction of a steady-state level. Whereas for IMR, although absolute inequality indicated a state of convergence, relative inequality continued to show a diverging trend. Non-parametric measures also affirm the hypothesis of a significant convergence for LEB and divergence for IMR over the period studied. Such findings illustrate divergent progress across different countries which may postpone a ‘Grand Convergence' in the global health scenario that was previously expected by 2035. Based on the speed of convergence, the estimated time to reach absolute convergence is: T = [LEB of 34.54 in 2015/(LEB of 34.54 in 2015*(2.2% per annum in 1950 to 2015/100)]. This suggests that with the current rate of convergence in global health status a ‘Grand Convergence’ is not possible before 2060 unless there are radical policy shifts. From a policy perspective, our findings also suggest that reducing avoidable mortality, improving access to basic amenities such as safe drinking water and sanitation in laggard countries will help them catching-up to life expectancy levels of developed countries.

The findings of this study in the context of existing literature in the field suggests that a significant amount of this divergent progress is possibly coming from within country socio-economic and regional inequalities among laggard countries which adversely affects their overall progress [1,21,30]. With economic growth, not everyone is getting rich at the same time, and not everyone is getting immediate access to the latest life-saving measures due to pre-existing social hierarchies. Deaton [3] describes it as “Growth, inequality, and catch up are two sides of the same coin. The dark side is what happens when the process is hijacked so that catch-up never comes. Powerful and wealthy elites have choked off demographic and health progress before, and they can do so again if they are allowed to undermine the institutions on which broad-based demographic and health progress depends”.

This study observes a notable progress made from the 1990's onwards in attaining global health goals especially with regards to reducing IMR, which also coincided with an increase in the number of countries left behind. The range of disparity across countries is critical in determining convergence. Even in 2010–15, the disparities were large. For instance, the LEB in North America is 79.2 years, while in Africa it is 59.5 years. Infant mortality rate (IMR) in Singapore and Japan is as low as 2 per 1000 live births, while it is 154 and 151 deaths per 1000 live births in Chad and the Central African Republic respectively [8]. Overall, in most countries, inequalities have been decreasing [31,32]. However, inequalities have been growing in a small but substantial number of countries [10, 32]. Previous research suggests that such a situation arises due to three main reasons: first, due to the diverse socio-economic progress, health care spending and health care-seeking pattern by the poor and rich in transition economies. With advances in new health care technologies, richer countries access them first compared to poorer countries. Within poorer countries, richer socio-economic classes access them first compared to their counterparts, which in turn leads to divergent progress [10, 32]. Second, due to the newly arising combinations of social, economic and political conditions, there is a rise in the burden of injury, impaired and mental health problems and deaths from violence, conflict, and war. There is also a rise in health risks consequent to large-scale environmental change and altered relations with the microbiological world caused by population pressure. That is, gains in longevity could be less smooth and less certain than what earlier notions of global convergence had suggested [14,15,16,29]. Third, the emergence of modern-day epidemics such as *Ebola*, *Swine* and *Bird flu* and epidemics like HIV/AIDs where poor in laggard countries are more vulnerable to exposure compared to leading countries, breaks the catch-up process from laggard to leading countries [16, 33].

### The added value of this study

The heterogeneous progress in mortality reduction and the unequal progress in life expectancy across different countries is a matter of great concern for establishing a fair, equitable and healthy society. In this context, this study provides evidence to answer the question: how far these differences are converging in the most recent decade, especially in the period of post-millennium development goals (MDGs) vis-à-vis previous decades. In particular, this study answers the question: did the emerging divergence in life expectancies after the late 1980s (as suggested in previous studies) [12, 17, 19], get replaced by re-convergence in the last decade, 2005–2015? Previous studies on the subject have reached diverse conclusions based on the methods they have used; while we used a comprehensive tool-kit of convergence metrics to test the convergence hypothesis on trends in world health status during the period 1950 to 2015.

## Conclusion and policy implications

Ensuring healthy lives and well-being for all is one of the goals of the newly formed Sustainable Development Goal (SDG’s). The findings of this study suggest that some countries have been progressing much faster than others. The uneven progress within and between the laggard countries in LEB and IMR during the period 1950 to 2015 has led to a heterogeneous scenario, although trends are moving towards a convergence point in the very recent period. Thus, we observe a trend of ‘rise and fall’ of global health inequalities, although fall of inequalities is not fast as expected due to poor progress in many developing countries which have missed MDGs. From a policy perspective, it is beneficial to observe the global trends and patterns of health indicators periodically since convergence or divergence processes may change over time. This study promotes the importance of using effective health monitoring tools such as convergence models when there are large socioeconomic and geographical disparities in the progress of health status. The policy analysts can use convergence measures as tools for health policy evaluations in laggard countries and suggest the trajectories to catch-up with advanced nations.

To fulfill the idea of ‘grand convergence' in health, we suggest that developing countries need to scale-up their health policies, make public health investments for health care and also invest in the adoption and innovation of new health technologies to catch-up with advanced countries. Similar suggestions were made by other studies. The *Lancet* Commission (prompted by the 20th anniversary of the 1993 World Development Report and supported by multiple organizations, the commission was formed to explore the case for investment in health and developed a new investment frame work to achieve dramatic health gains by 2035) revisited the case for investment in health and suggested that by 2035 nearly all countries could reach the frontier of feasibility, i.e., they could lessen their infectious, maternal, and child mortality rates down to those currently seen in the best performing middle and high-income countries. The primary concern in this context is a recognition of the most socio-economic vulnerable sub-populations in the laggard countries by national governments, so that new health policy and interventions can be streamlined according to their needs. Although not new but a repeatedly emerging theme from multiple studies [1,2,3,4,21] including the present study is that improving the availability of the cause of death information is critical for effective design and management of health problems in developing countries. Moreover, it is critical for policies to focus on financing of crucial health systems components (e.g., skilled health workers), creating comprehensive platforms for cost-effective clinical and preventive health interventions, international collective action to strengthen investments in health, delivery of improved health technologies, increased funding for health research and availability of reliable and comprehensive data on diseases that disproportionately affect low-income and middle-income countries.

## Supporting information

S1 Dataset Complete dataset used for computation (STATA). (DTA)

S1 Syntax Syntax and codes for the complete data (STATA DO). (DO)

## References

[1] Jamison DT, Summers LH, Alleyne G, Kenneth JA, Seth B, Binagwaho A, et al Salud global 2035: Un mundo convergiendo en el lapso de una generación. Salud Publica Mex. 2015;57(5):441–443. 10.1016/S0140-6736(13)62105-4 26545007

[2] Bloom DE, Canning D. Mortality traps and the dynamics of health transitions. Proc Natl Acad Sci. 2007;104(41):16044–16049. 10.1073/pnas.0702012104 17913890 PMC2042159

[3] Deaton A. The Great Escape: Health, Wealth, and the Origins of inequalityNo Title Princeton University Press; 2013.

[4] Naghavi M, Wang H, Lozano R, Davis A, Liang X, Zhou M, et al Global, regional, and national age-sex specific all-cause and cause-specific mortality for 240 causes of death, 1990–2013: A systematic analysis for the Global Burden of Disease Study 2013. Lancet. 2015;385(9963):117–171. 10.1016/S0140-6736(14)61682-2 25530442 PMC4340604

[5] Wang H, Liddell CA, Coates MM, Mooney MD, Levitz CE, SchumacherAE, et al Global, regional, and national levels of neonatal, infant, and under-5 mortality during 1990–2013: A systematic analysis for the Global Burden of Disease Study 2013. Lancet. 2014;384(9947):957–979. 10.1016/S0140-6736(14)60497-9 24797572 PMC4165626

[6] UNICEF, WHO. Levels & Trends in Child Mortality. Rep 2015 2015:1–34. 10.1371/journal.pone.0144443

[7] United Nations, Department of Economic and Social Affairs PD. No Title. World Population Prospects: The 2015 Revision, DVD Edition; 2015. https://esa.un.org/unpd/wpp/Download/Standard/Population/.

[8] United Nation, Department of Economic and Social Affairs PD. World Mortality Report [Highlights].; 2015. http://www.un.org/en/development/desa/population/publications/pdf/mortality/WMR2015/WMR2015_Highlights.pdf

[9] Margaret C Hogan, Alan D Lopez, Rafael Lozano, Christopher JL Murray, Mohsen Naghavi JKR. Building Momentum: Global Progress toward Reducing Maternal and Child Mortality.; 2010. http://www.healthdata.org/sites/default/files/files/policy_report/2010/Building_Momentum/IHME_BuildingMomentum_FullReport.pdf.

[10] Wagstaff A, Bredenkamp C, Buisman LR. Progress Toward the Health MDGs Are the Poor Being Left Behind? 2014;(May):1–37. http://documents.worldbank.org/curated/en/138361468338684218/pdf/WPS6894.pdf

[11] Marmot M. The Health Gap: The Challenge of an Unequal World: The argument. Int J Epidemiol. 2017;46(4):1312–1318. 10.1093/ije/dyx163 28938756 PMC5837404

[12] Dorius SF. Global demographic convergence? A reconsideration of changing intercountry inequality in fertility. Popul Dev Rev. 2008;34(3):519–537. 10.1111/j.1728-4457.2008.00235.x

[13] Institute for Health Metrics and Evaluation (IHME). Financing Global Health 2016 Development Assistance, Public and Private Health Spending for the Pursuit of Universal Health Coverage.; 2017. http://www.healthdata.org/policy-report/financing-global-health-2016-development-assistance-public-and-private-health-spending

[14] McMichael AJ, McKee M, Shkolnikov V, Valkonen T. Mortality trends and setbacks: global convergence or divergence? Lancet. 2004;363(9415):1155–1159. 10.1016/S0140-6736(04)15902-3 15064037

[15] Moser K, Shkolnikov V, Leon DA. World mortality 1950–2000: Divergence replaces convergence from the late 1980s. Bull World Health Organ. 2005;83(3):202–209. doi: /S0042-96862005000300013 15798844 PMC2624202

[16] Neumayer E. HIV/AIDS and Cross-National Convergence in Life Expectancy. 2004;30(December):727–742. http://www.jstor.org/stable/3657336

[17] Cole MA, Neumayer E. The pitfalls of convergence analysis: Is the income gap really widening? Appl Econ Lett. 2003;10(6):355–357. 10.1080/1350485032000072361

[18] Wilson C. Understanding global demographic convergence since 1950. Popul Dev Rev. 2011;37(2):375–388. 10.1111/j.1728-4457.2011.00415.x

[19] Council P, Review D. On the Scale of Global Demographic Convergence 1950–2000 Author (s): Chris Wilson Source: Population and Development Review, Vol. 27, No. 1 (Mar., 2001), pp. 155–171 Published by: Population Council Stable URL: http://www.jstor.org/stable/269. 2017;27(1):155–171. 10.1111/j.1728-4457.2001.00155.x 18589488

[20] Dybul M. A grand convergence and a historic opportunity. Lancet. 2013;382(9908):e38–e39. 10.1016/S0140-6736(13)62344-2 24309479

[21] Goli S, Arokiasamy P. Maternal and child mortality indicators across 187 countries of the world: Converging or diverging. Glob Public Health. 2014;9(3):342–360. 10.1080/17441692.2014.890237 24593038

[22] Jamison DT, Summers LH, Alleyne G, Arrow KJ, Berkley S, Binagwaho A, et al Global health 2035: a world converging within a generation. Lancet. 2013;382: p1898–1955. 10.1016/S0140-6736(13)62105-4 24309475

[23] Shkolnikov VM, Andreev EE, Begun AZ. Gini coefficient as a life table function: Computation from discrete data, decomposition of differences and empirical examples. Demogr Res. 2003;8:305–357. 10.4054/DemRes.2003.8.11

[24] Barro R, Sala-i-Martin X. Convergence. J Polit Econ. 1992;100(2):223–252.

[25] Young AT, Higgins MJ, Levy D. Sigma convergence versus beta convergence: Evidence from U.S. county-level data. J Money, Credit Bank. 2008;40(5):1083–1093. 10.1111/j.1538-4616.2008.00148.x

[26] Strulik H, Vollmer S. The fertility transition around the world. J Popul Econ. 2015;28(1):31–44. 10.1007/s00148-013-0496-2

[27] Quah. Empirical cross section dynamics in economic growth. Eur Econ Rev. 1993;37:426–434. https://www.isid.ac.in/~tridip/Teaching/DevEco/Readings/02Convergence/06Quah-EER1993.pdf

[28] Wang Y. A nonparametric analysis of the personal income distribution across the provinces and states in the US and Canada. Reg Sect Econ Stud. 2004;4:5–24. http://www.usc.es/economet/reviews/eers411.pdf

[29] Vallin J, Meslé F. Convergences and divergences in mortality. A new approach to health transition. Demogr Res. 2004;10(SUPPL. 2):11–44. 10.4054/DemRes.2004.S2.2

[30] Wagstaff A. Inequalities in health in developing countries: Swimming against the tide? World Bank Policy Res Work Pap. 2002;2795(February 2002). https://openknowledge.worldbank.org/bitstream/handle/10986/14858/multi0page.pdf?sequence=1&isAllowed=y

[31] Victora CG, Barros AJD, Malpica-Llanos T, Walker N. How within-country inequalities and co-coverage may affect LiST estimates of lives saved by scaling up interventions. BMC Public Health. 2013;13(Suppl 3):S24 10.1186/1471-2458-13-S3-S24 24564259 PMC3847580

[32] Gwatkin DR. Trends in health inequalities in developing countries. Lancet Glob Heal. 2017;5(4):e371–e372. 10.1016/S2214-109X(17)30080-3 28238720

[33] Daher M. World report on violence and health. J Med Liban. 2002;51(2):59–63. 10.1136/ip.9.1.93 15298158

